# Pharmacists’ Understanding and Attitudes Toward Pharmaceutical Care in Saudi Arabia

**DOI:** 10.7759/cureus.51255

**Published:** 2023-12-28

**Authors:** Hanan S Alromaih, Waad A Alanzi, Abdulmajeed Alqasoumi, Ahmer H Mirza

**Affiliations:** 1 Department of Pharmaceutics, College of Pharmacy, Qassim University, Buraydah, SAU; 2 Department of Toxicology, College of Pharmacy, Qassim University, Buraydah, SAU; 3 Department of Pharmacy Practice, College of Pharmacy, Qassim University, Buraydah, SAU; 4 Department of Pharmacy, School of Applied Science, University of Huddersfield, Huddersfield, GBR

**Keywords:** saudi arabia, knowledge, attitudes, pharmaceutical care, hospital

## Abstract

Background

It has been proposed that pharmacy practice moves from product-focused to patient-focused. The major goals of pharmaceutical care are to improve the patient’s quality of life, approve the safety of drug therapy, and seek to enhance the quality of life related to the patient’s health within reasonable financial constraints. One of the biggest issues facing Saudi Arabia’s healthcare system is including a pharmacist in the healthcare team who uses the most up-to-date pharmaceutical services and cares for the patient with medical and nursing care. This study aims to assess the opinions and knowledge concerning pharmaceutical care of hospital pharmacists working in three major governmental hospitals in the Al-Qassim region.

Methodology

A descriptive cross-sectional survey using an anonymous, structured, validated, and pilot-tested questionnaire of hospital pharmacists was utilized to address the study’s objectives. The data were collected in four weeks between November and December 2021 from three major governmental hospitals in the Al-Qassim region. Data were analyzed using the SPSS software version 24 (IBM Corp., Armonk, NY, USA), and the associations between variables were evaluated.

Results

The survey was completed by 130 of the 160 pharmacists during the four-week study period (the overall response rate was 81%). The majority of those who responded were Saudi males, and only 19 (14.6%) participants had more than 15 years of practice. The majority of pharmacists had favorable opinions on providing pharmaceutical care. Overall, 122 (93.9%) respondents knew what pharmaceutical care meant. Most pharmacists (120, 92.3%) agreed to always counsel the patient on how to use their medications. Nearly half of the pharmacists (58, 45.0%) lacked knowledge about the clinical disease states, and 108 (83.0%) pharmacists knew how to obtain information.

Conclusions

Pharmacists reported a positive attitude regarding pharmaceutical treatment. Awareness and attitudes concerning pharmaceutical treatment were associated with a variety of demographic parameters, including gender, years of experience, and place of employment, to name a few. They reported that they were willing but concerned about their clinical expertise and ability to communicate effectively. Therefore, pharmacists should be permitted to enroll in programs that teach them how to provide pharmacological care.

## Introduction

Throughout the world, pharmacies are sites where people can get help managing their illnesses with medication and accept help from the healthcare system [1]. Patients’ quality of life can be improved by pharmaceutical care, which is a philosophy of practice in which a pharmacist prescribes drugs to achieve particular outcomes. Pharmacists collaborate with other healthcare professionals and patients to develop, implement, and track a treatment plan to diagnose, treat, and prevent medication-related problems [2]. Pharmacists are often the first point of contact for patients who are unable to reach a doctor directly, and their specialized duties and activities in the healthcare system are evolving from old-style responsibilities such as dispensing and formulating drugs to increased attention on patient care. The practice of community pharmacy differs from country to country as community pharmacists are the closest healthcare experts to patients for their drugs and health-related advice [1-3].

Improvements are being made to pharmacy education in Saudi Arabia to ensure it matches international standards. The expansion of pharmacy schools in the country and the requirement for clinical practitioners are the primary factors behind this trend. The number of pharmacy schools in Saudi Arabia increased from two in 2002 to roughly 30 by 2012. However, most of these programs do not include specific university courses focusing on community pharmacy. Therefore, students still lack basic awareness of this scenario [3].

Compared to other localities, pharmacists in Al-Qassim appear to offer a wide range of pharmaceutical care facilities that might serve as a foundation for proper pharmaceutical care. Owing to competition among community pharmacies, pharmacists may strive to provide the best possible patient care [4].

Pharmaceutical care has been described as “the responsible provision of medication therapy for the objective of producing consequences that enhance a patient’s quality of life.” There are three primary responsibilities that pharmacists should collaborate with other healthcare providers on. These include identifying and recognizing problems with medication therapy, resolving actual issues relating to pharmacological therapy, and preventing probable drug therapy difficulties [5,6].

Most Saudi pharmacy graduates work in hospitals or government agencies, and the number of pharmacy graduates employed in community pharmacies is quite low, with many new graduates unsure of their career paths in this sector. Pharmaceutical care is a relatively new service being adopted in Saudi Arabia, primarily by hospital pharmacists [3,4]. Young Saudi pharmacists are becoming increasingly involved in basic healthcare, which is to be expected given the country’s soaring number of pharmacy school graduates, which considerably exceeds the capacity of local hospitals. In the absence of a trained pharmacist, patients in private clinics and hospitals are referred to their local community pharmacy to obtain their prescribed prescriptions [7,8].

To our knowledge, no study has been performed to better understand the types and levels of services currently provided by pharmacists in Saudi Arabia working in hospitals [9]. In Qatar, the pharmacy profession, except for a few patient-centered cognitive therapies, is primarily focused on dispensing medications, and pharmacy practice is still in its infancy. One of the missions of the College of Pharmacy at Qatar University is to prepare pharmacy students to offer the best computers and improve healthcare outcomes [5]. In Poland, the average time for a medical consultation is reduced, the medical examination of the patient often follows only the issuance of a prescription and the provision of any evasive information, and the provision of a pharmacy practice appears to be crucial [1]. Only one study in Kuwait of 80 hospital pharmacists in four public hospitals determined the level of application of pharmaceutical care activities, willingness to change practice, and challenges that may delay the application of pharmaceutical care, with the study revealing the lack of understanding of pharmaceutical care practice among pharmacists [2]. The lack of a reimbursement system was cited as the most significant impediment in the United States, whereas uncertainty about pharmacists’ attitudes toward pharmaceutical care was found to hinder the advancement of the pharmaceutical care service and pharmacists’ professional roles in Iran and Pakistan [7].

Even though research is essential regardless of the field of practice to increase professional growth, practice, and education in Nigeria and other developing countries, patient-oriented pharmacy practice research in hospitals and communities is uncommon [10,11]. According to a study, hospital pharmacists in Nigeria have a poor attitude toward pharmaceutical care [10,11]. In Jordan, with the introduction of pharmaceutical care units into university pharmacy curricula and the opening of the Doctor of Pharmacy and masters programs in clinical pharmacy, the level of knowledge of pharmaceutical care among Jordanian pharmacists was suitable; however, the application of the service was restricted [12].

The practice of pharmacy in hospitals is more developed than community pharmacy practice in Saudi Arabia. The way it focuses on profits of professional services, which results in being below the expectations of the health authority, makes the contribution of primary health center pharmacists in Saudi Arabia vital [7,8],

The quality of pharmaceutical care should be evaluated both from an individualist perspective, which emphasizes patient values, expectations, and desires, and a societal perspective, which determines the utility of pharmaceutical care regarding our healthcare system. Patients’ opinions of the quality and value of care are critical, as these may influence their willingness to participate actively in healthcare services (e.g., ask questions, keep appointments, comply with medical advice and treatment regimens) [13].

The evolution of pharmacy practice has been marked by a series of stages, namely, manufacturing, compounding, distribution, clinical pharmacy, and pharmaceutical care. Rather than a sequence of dramatic changes, this process has been defined as an uneven adoption of a new practice model as prospects for its presence arise [14]. Although pharmacy has lost its apothecary role, it has not yet regained its former prominence in medical care. It would not be enough to deliver the right drug or give advanced pharmaceutical services; neither will it be enough to develop new technical features [15].

Pharmaceutical care is not fully adopted in any health system, but several developed countries have created promising projects [16]. Pharmacists from various backgrounds and environments must identify their challenges and prioritize the issues that must be addressed first. For example, serious scarcity of resources appears to be the most significant obstacles to oversome in developing countries [17]. Predefined tasks such as drug order entry occupying pharmacists’ time, personnel lacking clinical knowledge and communication skills, a lack of pharmacy technicians to assist with dispensing duties, pharmacists lacking confidence, and pharmacists being absent from patient care areas hindering pharmacist-patient interaction are common barriers. Furthermore, one of the most significant impediments to its practice has been pharmacists’ poor views toward providing pharmaceutical care [18]. Hence, this study aims to evaluate the role of pharmacists in knowledge and delivering pharmaceutical care in major governmental hospitals and determine hospital pharmacists’ understanding, attitudes, and barriers in all departments that limit the provision of pharmaceutical care.

## Materials and methods

Study design, setting, and population

A descriptive cross-sectional survey was conducted using an anonymous, structured, validated, and pilot-tested questionnaire of hospital pharmacists to address the study’s objectives. The data were collected from King Fahad Specialist Hospital, Maternity and Children’s Hospital, and Buraydah Central Hospital, which are major governmental hospitals in the Al-Qassim region. Pharmacists who agreed to participate in the study were given an overview of the study objectives and asked if they agreed to participate. All hospital pharmacists and pharmacy doctors in King Fahad Specialist Hospital, Maternity and Children’s Hospital, and Buraydah Central Hospital in the Al-Qassim region who were eligible were contacted. Pharmacist technicians, community pharmacists, and hospital pharmacists in other areas were excluded from this study. A total of 130 participants were included, and data were collected in the four weeks between November and December 2021.

Development and survey validation

In this study, a self-administered questionnaire as well as online Google Forms was used. This survey was designed in English to measure pharmacists’ understanding and attitudes toward pharmaceutical care and the barriers perceived in providing pharmaceutical care. The survey questions were collected from several previous studies as well as self-made questions. The data collection tool was validated by a panel of two pharmaceutical care professors expert in survey methodology. Face validation was done with open discussions to ensure its content validity and applicability. The reliability of the questionnaire was assessed by a pilot study involving 15 participants, resulting in a reliability coefficient (Cronbach’s alpha) of 0.76.

The survey had 20 close-ended questions that took about 7-10 minutes to complete. It included questions about pharmacists’ opinions regarding pharmaceutical care and perceived impediments to pharmaceutical care, as well as their sociodemographic characteristics. Sociodemographic characteristics included nationality, gender, age group, and years of practice. A five-point Likert scale (1 = strongly disagree, 5 = strongly agree) was used to gauge the amount to which the pharmacists agreed with 16 statements relating to pharmaceutical care to examine their knowledge and attitudes toward pharmaceutical care. These statements measured three constructs, namely, pharmacists’ understanding and attitudes toward pharmaceutical care (Q1-6), pharmacists’ duty (Q7-11), and perceived barriers (Q12-16).

Execution of survey and confidentiality

A self-administered survey was distributed to hospital pharmacists after obtaining ethical approval and hospital and pharmacy management consent. Each hospital was given two weeks, and after the first week, a reminder visit was done. The online survey was done via Google Survey and was sent via email as well as a link to hospital pharmacist practitioners who we could not cover via the paper form. A total of 87 surveys were collected in person and 43 were collected via Google Forms, with a total of 130 participants. The survey was conducted anonymously to eliminate any potential for bias and to ensure participant confidentiality. The investigators did not record any information regarding the participants’ identities.

Data analysis

Data from both paper and internet surveys were entered into SPSS software version 24 (IBM Corp., Armonk, NY, USA). Frequency and percentage were used for qualitative data. If incomplete surveys had basic demographic information and partial replies to any of the questions, they were considered in the study. As a result, the number of respondents who answered each question differed. The five Likert scores (1-5) of the relevant items were added to calculate the score for each item: pharmacists’ understanding and attitudes toward pharmaceutical care statements (Q1-6), pharmacists’ duty statements (Q7-11), and perceived barriers statements (Q12-16). Positive responses were considered as scores above 3. For pharmacists’ understanding and attitudes toward pharmaceutical care, the highest achievable score was 30, and 6 was the lowest. The highest achievable score for pharmacists’ duties was 25, and 5 was the lowest. For perceived barriers, the highest achievable score was 25, and 5 was the lowest.

Ethical considerations

This study was evaluated and approved by the relevant ethics committee and carried out in compliance with the ethical principles of Saudi Arabia. Participants’ participation in the study was completely voluntary and posed no risk to them. Pharmacists’ who answered the survey were deemed to have granted their agreement to participate in the research. Ethical approval was obtained from the Qassim Research Committee (approval number: 14430711868).

## Results

The survey was completed by 130 of the 160 pharmacists during the four-week study period (achieved overall response rate was 81%).

Table 1 summarizes the general characteristics of respondents. The majority of those who responded were Saudi (127, 97.7%) and males (92, 70.8%). Regarding age, 71 (54.6%) of the pharmacists were between the ages of 31 and 41 years, 36 (27.0%) were 23-30 years old, 19 (14.6%) were 42-52, and four participants (3.1%) were 53 years old or more. The majority (44, 33.8%) had less than five years of pharmacy practice, the rest had 6-10 years of experience (36, 27.7%), and 11-15 years of experience (22, 16.9%), while only 19 (14.6%) had more than 15 years of practice.

**Table 1:** General characteristics of the research participants.

Parameter		Frequency	Percentage
Sex	Male	92	70.8%
	Female	37	28.5%
Nationality	Saudi	127	97.7%
	Non-Saudi	3	2.3%
Age	23–30 years	36	27.0%
	31–41 years	71	54.6%
	42–52 years	19	14.6%
	52–60 years	4	3.1%
Years of practice	1–5	44	33.8%
	6–10	36	27.7%
	11–15	22	16.9%
	More than 15	19	14.6%

Table 2 shows hospital pharmacists’ attitudes toward each pharmaceutical care item. Generally, pharmacists were passionate about providing pharmaceutical care. A total of 122 respondents (93.9%) knew what pharmaceutical care meant. Overall, 100 (84.5%) believed that a pharmacist’s major job in a healthcare context is to provide pharmaceutical care as well as to prevent and resolve medication-related issues, and 112 (86.8%) thought that the practice of pharmaceutical care is valuable. Further, 63 (48.9%) pharmacists thought that pharmacists are involved in the therapeutic plan for inpatients only, and 54 (41.9%) disagreed with that. On the other hand, 75 (57.7%) felt that delivering pharmaceutical care takes too much time and effort.

**Table 2:** Understanding and attitudes of pharmaceutical care among the research participants.

Parameter	Value; n (%)				
	Strongly agree	Agree	Neutral	Disagree	Strongly disagree
I know what pharmaceutical care means	85 (65.4)	37 (28.5)	5 (3.8)	2 (1.5)	1 (0.8)
Pharmacists are involved in the therapeutic plan for inpatients only	26 (20.2)	37 (28.7)	12 (9.3)	24 (18.6)	30 (23.3)
The primary responsibility of pharmacists in a healthcare setting should be to prevent and solve medication-related problems	34 (33.3)	66 (51.2)	18 (14.0)	2 (1.6)	0 (0.0)
Pharmacists’ primary responsibility should be to practice pharmaceutical care	39 (30.5)	69 (53.9)	12 (9.4)	6 (4.7)	2 (1.6)
I think the practice of pharmaceutical care is valuable	57 (44.2)	55 (42.6)	12 (9.3)	4 (3.1)	1 (0.8)
Providing pharmaceutical care takes too much time and effort	26 (20.0)	49 (37.7)	32 (24.6)	19 (14.6)	4 (3.1)

Table 3 presents the pharmacists in the hospital pharmacy who reported performing pharmaceutical care-related services. In total, 102 (78.4%) participants agreed that they always counsel the patient on why they were prescribed particular medications. Most pharmacists (120, 92.3%) agreed to always counsel the patient on how to use their medications. Further, 104 (81.2%) also agreed that they always counsel the patient on the side effects of their medications. Almost two-thirds (100, 76.9%) agreed to always counsel the patient on drug and/or food interactions. Finally, about 105 (81.4%) agreed to always inform the patient on how to store their medications.

**Table 3:** Pharmacist duties and responsibilities among the research participants.

Parameter	Value; n (%)				
	Strongly agree	Agree	Neutral	Disagree	Strongly disagree
I always counsel the patient on why they were prescribed particular medications	45 (34.6)	57 (43.8)	20 (15.4)	6 (4.6)	2 (1.5)
I always counsel the patient on how to use their medications	74 (56.9)	46 (35.4)	7 (5.4)	2 (1.5)	1 (0.8)
I always counsel the patient on the side effects of the medications	41 (32.0)	63 (49.2)	19 (14.8)	5 (3.9)	0 (0.0)
I always counsel the patient on drug and/or food interactions	49 (37.7)	51 (39.2)	25 (19.2)	4 (3.1)	1 (0.8)
I always inform the patient on how to store their medications	58 (45.0)	47 (36.4)	20 (15.5)	4 (3.1)	0 (0.0)

Table 4 summarizes the practice of pharmaceutical care in hospital pharmacies affected by various obstacles. Nearly half the pharmacists (58, 45.0%) lacked knowledge about the clinical disease states. In total, 52 (47.8%) said that there is not much communication with physicians. Almost two-thirds (91, 70%) said that patients are always in a rush when counseling. Further, 108 (83%) knew how to reach obtain information. Lastly, more than half (83, 63.8%) agreed that there is a sufficient number of workers to carry the workload.

**Table 4:** Barriers faced during pharmaceutical care among the research participants.

Parameter	Value; n (%)				
	Strongly agree	Agree	Neutral	Disagree	Strongly disagree
I lack the knowledge about the clinical disease states	17 (13.2)	41 (31.8)	39 (30.2)	22 (17.7)	10 (7.8)
There is not much communication with physicians	17 (13.1)	45 (34.6)	30 (23.1)	23 (17.7)	15 (11.5)
Patients are always in a rush when counseling	28 (21.5)	63 (48.5)	31 (23.8)	7 (5.4)	1 (0.8)
I know how to obtain information	51 (39.2)	57 (43.8)	19 (14.6)	2 (1.5)	1 (0.8)
There is a sufficient number of workers to carry the workload	26 (20.0)	57 (43.8)	23 (17.7)	17 (13.1)	7 (5.4)

## Discussion

There has been a shift in the responsibility of the pharmacist in hospital pharmacies to provide improved pharmacological care services and better patient counseling, vaccination, as well as drug administration [3]. However, countries differ in their conceptions of the best ways to provide pharmacological treatment [3]. Pharmacists in Saudi Arabia are becoming more involved in care delivery related to patients than product care as a result of a shift in pharmacy practice over time [3].

Among the 130 pharmacists who participated in our study, the majority were Saudi males. Pharmacy professionals typically range in age from 31 to 41 years old (71, 54.6%). In a survey of pharmacists’ opinions regarding pharmaceutical care, the majority (44, 33.8%) have fewer than five years of experience in pharmacy practice, although the majority (122, 93.9%) understood what pharmaceutical care entails. Preventing and solving medication-linked issues is a primary role of pharmacists in the healthcare system, and the majority of pharmacists (100, 84.5%) agreed. The majority (112, 86.8%) also felt that pharmacists’ practice of pharmaceutical care is essential. On the other hand, 75 (57.7%) respondents said that providing pharmacological treatment was time-consuming and difficult. An alternative study indicated that pharmacists who work in hospital pharmacies (96, 42.9%) are more likely to be under 30 years old (110, 49.1 %) and to have fewer than five years of pharmacy experience (110, 49.1%). Moreover, individuals who have only a bachelor’s degree were estimated at 197 (88%) [4]. A small sample of the general community in Qatar was surveyed, and it was noted that “the public has a poor awareness of the pharmacist’s role in connection to medical assessment, drug treatment management, and drug information dissemination.” However, research has shown that pharmacist-provided pharmaceutical practice services are effective in terms of clinical, humanistic, and economic results in various illnesses [5].

The vast majority of pharmacists (102, 78.4%) in our study stated that they always explain the rationale for prescribing a particular drug to patients. Overall, 120 (92.3%) pharmacists said they always offer advice to patients on how to properly utilize their prescriptions. Further, 104 (81%) pharmacists also stated that they always inform patients about the potential negative effects of the medications they prescribe. Roughly 100 (76.9%) agreed that patients should always be informed about possible drug-food interactions. About 105 (81.4%) respondents agreed that patients should always be informed of proper storage methods for their drugs. In another study, 189 (84.4%) pharmacists believed that drug history is of little or no importance. Others noted that nine (4%) pharmacists provided information on drug side effects, and four (2%) had an understanding of drug storage. Patients were notified about medicine and/or food interactions by four (2%) pharmacists [4].

Pharmacists in our study were found to have a lack of understanding of clinical illnesses in nearly half of those surveyed (58, 45.0%). Some (52, 47.8%) also stated that there is little to no communication between patients and doctors. A shocking 91 (70.0%) respondents claimed that their patients often rush through their sessions with them. Overall, 108 (83%) pharmacists could find information. Finally, more than half of respondents (83, 63.8%) felt that the number of employees is adequate to handle the work. According to the findings of this research, however, 112 (50.0%) pharmacists lacked clinical knowledge about disease conditions, 107 (47.8%) lacked technical knowledge about providing pharmaceutical treatment, 111 (49.6%) lacked suitable numbers of assistants, and 199 (88.8%) had increasing workload [4].

Expanded specialized jobs necessitate further education and training for pharmacists. Pharmacists should be given particular training or educational programs to improve their ability to provide pharmaceutical care. To enhance patient care in addition to the partnership between pharmacists and physicians they should work together to develop interprofessional continuing education programs in therapies [7].

## Conclusions

Pharmaceutical care is widely considered to be the responsibility of pharmacists by the vast majority of pharmacists. Patients in a hospital pharmacy can expect a wide range of services connected to pharmaceutical care, including taking drug histories, educating on how to use medications, and getting information about the interactions between medications as well as nourishment. A lack of clinical understanding of disease cases, a shortage of technical expertise in delivering pharmaceutical care, a shortage of support workers, and an overburdening workload are among the biggest obstacles pharmacists face when providing pharmaceutical treatment in community pharmacies.

Saudi Arabia is not different when it comes to pharmaceutical care. Pharmacists in different nations appear to offer similar services and identify similar barriers. Further research with a large sample size and more hospitals is required to support these findings.
